# Long Noncoding RNAs CARMN, LUCAT1, SMILR, and MALAT1 in Thoracic Aortic Aneurysm: Validation of Biomarkers in Clinical Samples

**DOI:** 10.1155/2020/8521899

**Published:** 2020-06-17

**Authors:** Vaiva Patamsytė, Giedrius Žukovas, Dovydas Gečys, Diana Žaliaduonytė, Povilas Jakuška, Rimantas Benetis, Vaiva Lesauskaitė

**Affiliations:** ^1^Institute of Cardiology, Lithuanian University of Health Sciences, Sukilėlių pr. 15, Kaunas LT-50103, Lithuania; ^2^Department of Cardiac, Thoracic and Vascular Surgery, Lithuanian University of Health Sciences, Eivenių g. 2, Kaunas LT-50161, Lithuania; ^3^Department of Cardiology, Lithuanian University of Health Sciences, Eivenių g. 2, Kaunas LT-50161, Lithuania

## Abstract

**Materials and Methods:**

Relative expression of lncRNAs CARMN, LUCAT1, SMILR, and MALAT1 was tested in clinical aortic tissue and blood plasma samples from TAA and non-TAA patients using the qRT-PCR method. The Mann–Whitney *U* test was used to compare *Δ*Ct values between the study groups. ROC curve analysis was performed to evaluate the diagnostic value of plasma lncRNAs.

**Results:**

We found significantly reduced CARMN (*p* = 0.033) and LUCAT1 (*p* = 0.009) expression in aortic tissue samples from TAA patients. Relative expression of MALAT1 (*p* = 0.117) and SMILR (*p* = 0.610) did not differ in aortic tissue between the TAA and non-TAA groups. Expression of both LUCAT1 and SMILR was significantly decreased in TAA patients' blood plasma compared to controls (*p* = 0.018 and *p* = 0.032, respectively). However, only LUCAT1 showed the ability to discriminate aneurysmal disease in patients' blood plasma (AUC = 0.654, 95%CI = 0.534‐0.775, *p* = 0.018).

**Conclusions:**

We have shown that the expression of lncRNAs CARMN and LUCAT1 is reduced in dilated aortic tissue and that the LUCAT1 and SMILR expression is lower in the blood plasma of TAA patients. Decreased LUCAT1 expression in TAA patients' blood plasma may have diagnostic potential in discriminating patients with TAA.

## 1. Introduction

Thoracic aortic aneurysm (TAA) is mainly an asymptomatic disease with an increasing incidence rate [1]. Biomarker research has long been a focus for the development of an effective clinical screening for TAA [2]. Sequencing technologies lead to identification of various noncoding RNAs which are considered key players in the regulation of biological processes. A class of small noncoding RNAs called microRNAs (miRNAs) is extensively studied in the development of cardiovascular diseases (CVDs) as potential biomarkers and therapeutic targets [3]. Intriguing information begins to emerge regarding the role of long noncoding RNAs (lncRNAs), but these studies are still limited, and specific contributions of lncRNAs to the disease development and progression remain unexplored [4]. lncRNAs are transcripts larger than 200 nt and exert different cellular function depending on their localization in the nucleus or cytoplasm [5, 6]. TAA shows common histological features such as the vascular smooth muscle cell (VSMC) phenotype switch from contractile to synthetic and extracellular matrix remodelling [7]. Several mechanisms are revealed by which lncRNAs control VSMC migration [8], proliferation [9], and angiotensin II (Ang II) signalling system [10]. Due to their regulatory capabilities, lncRNAs might play an important role in aortic aneurysmal disease [11].

Circulating lncRNAs show a diagnostic potential in coronary artery disease [12, 13], acute myocardial infarction [14], diabetic cardiomyopathy [15], and chronic heart failure [16]. A study on the rat model has reported increased expression of specific lncRNAs in both plasma and cardiac samples during acute myocardial infarction [14]. Recent *in vitro* studies identify lncRNAs, associated with the VSMC phenotypic state, namely, metastasis-associated lung adenocarcinoma transcript 1 (MALAT1) [17], smooth muscle-induced lncRNA (SMILR), cardiac mesoderm enhancer-associated lncRNA (CARMN), and lung cancer-associated transcript 1 (LUCAT1) [18]. Using clinical samples, we test if these lncRNAs are differentially expressed in the aortic wall and blood plasma of TAA patients and individuals without aortic dilatation. This information will help to assess the potential of MALAT1, SMILR, CARMN, and LUCAT1 to be used as biomarkers for the formation of TAA.

## 2. Materials and Methods

### 2.1. Study Subjects

Study subjects were recruited at the Hospital of Lithuanian University of Health Sciences, Department of Cardiology and Department of Cardiac, Thoracic and Vascular Surgery during the period of 2016-2019. Aortic tissue samples (*N* = 24) and blood plasma samples (*N* = 40) were obtained from sporadic TAA patients, who were diagnosed with aortic aneurysm in accordance with 2014 ESC guidelines on the diagnosis and treatment of aortic disease [19]. Aortic tissue samples of the non-TAA group (*N* = 42) consisted of heart donors (*N* = 5) without a history of cardiovascular pathology and patients (*N* = 37) who had an isolated coronary artery bypass procedure (CABG) without aortic dilation. Non-TAA blood plasma samples (*N* = 53) were collected from study subjects, who did not have a diagnosis of TAA. All study subjects except heart donors had two-dimensional thoracic echocardiography. Our study excluded cases of severe atherosclerosis of the ascending aorta (*N* = 3), aortitis (*N* = 4), and genetic syndromes (*N* = 2, both Marfan). The study was approved by the Kaunas Regional Biomedical Research Ethics Committee (Nr. P2-BE-2-12/2012) in accordance with the Declaration of Helsinki.

### 2.2. Sample Collection

Tissue samples from TAA patients were collected from convexity of the ascending aorta. Segments of 5 × 5 mm in size were cut out from the outer curvature. Aortic specimens from CABG patients were taken from the ascending aorta at the site of proximal bypass anastomosis (punch biopsies). Ascending aorta samples from donors were taken during the heart transplantation procedure. All tissue samples were immediately put into RNAlater™ (Thermo Fisher Scientific, Lithuania) stabilization solution, kept at +4°C overnight, and later stored at −80°C. Whole blood samples from TAA and non-TAA study subjects were collected into 3 ml Venosafe™ vacutainer tubes with K2EDTA (Terumo Europe, Belgium) before heparinization. Blood plasma was prepared within an hour of phlebotomy procedure by centrifugation at 1,900 g, for 10 min. Plasma samples were stored at −80°C.

### 2.3. RNA Extraction and Quantification

Aortic tissue samples were frozen with liquid nitrogen and manually ground for total RNA extraction using a mirVana™ miRNA Isolation Kit (Thermo Fisher Scientific, Lithuania) and Acid Phenol : CHCl3 premix (Ambion, USA) according to the manufacturer's protocol. RNase Inhibitor (Applied Biosystems, USA) was added to a lysis buffer to prevent RNA degradation. RNA concentration and integrity were assessed using a NanoDrop 2000 Spectrophotometer (Thermo Scientific) and RNA 6000 Nano Kit (Agilent Technologies, USA) on a 2100 Bioanalyzer System (Agilent). RNA samples were treated with a DNase TURBO DNA-free™ Kit (Thermo Fisher Scientific, Lithuania) to eliminate any DNA contamination. A miRNeasy Serum/Plasma Kit (QIAGEN, Germany) was used for total RNA extraction from plasma samples, according to the manufacturer's protocol. Reverse transcription for lncRNA expression analysis was done with High Capacity RNA-to-cDNA Kit (Applied Biosystems, USA).

Gene expression experiments were done according to the manufacturer's protocol on an ABI 7900HT Fast Real Time PCR System (Applied Biosystems) using TaqMan® Fast Advanced Master Mix (Applied Biosystems, USA) and TaqMan Assays. Expression of lncRNAs was evaluated using TaqMan® Gene Expression Assays for MALAT1 (assay ID Hs00273907_s1), CARMN (assay ID Hs04402463_m1), LUCAT1 (assay ID Hs00884761_s1), and a custom-made SMILR assay with forward primer sequence 5′-GGATATGAATTGTAATGGCCAGAGCAT-3′, reverse primer sequence 5′-GAATTCAGTCTTGGTTCCCTAAAATGG-3′, and a reporter dye FAM with sequence CTGTGAGATGAAAACTC. Expression level of glyceraldehyde 3-phosphate dehydrogenase (GAPDH) [15] (assay ID Hs99999905_m1), was used as an endogenous control for lncRNA data normalisation. All *Δ*Ct values were calculated using ExpressionSuite v1.2 Software (Thermo Fisher Scientific) and log-transformed.

### 2.4. Statistical Analysis

Data normality was checked using a Kolmogorov-Smirnov test. A nonparametric Mann–Whitney *U* test was used to compare log-transformed *Δ*Ct values of lncRNAs between the study groups. Differences in clinical characteristics were calculated using Mann–Whitney *U* test and chi-squared test. Receiver operating characteristic (ROC) curve analysis was performed to evaluate the diagnostic value of plasma lncRNAs in TAA and non-TAA patients. GraphPad Prism 8 software (San Diego, CA, USA) was used to construct plots and perform calculations.

## 3. Results

**Figure 1:**
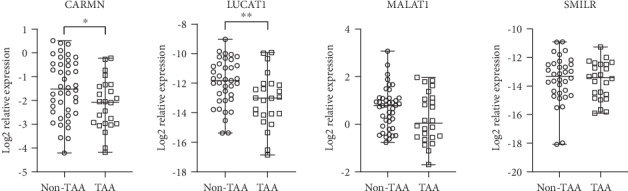
Relative expression of CARMN, LUCAT1, MALAT1, and SMILR in the human aortic wall of non-TAA and TAA patients. Whiskers indicate minimum and maximum values; horizontal lines represent median values. ∗ indicates *p* < 0.05; ∗∗ indicates *p* < 0.01.

Clinical characteristics of patients in all study groups are summarized in Table 1. We did not find an association between lncRNA expression profiles and presence of aortic valve insufficiency and hypertension (*p* > 0.05). Only LUCAT1 expression in the aortic wall was significantly reduced in the study subjects with a bicuspid aortic valve (BAV) compared to tricuspid aortic valve (TAV) patients (*p* = 0.004). Relative expression profiles of CARMN, LUCAT1, MALAT1, and SMILR in aortic wall specimens of non-TAA and TAA patients are shown in Figure 1. Both CARMN (*p* = 0.033) and LUCAT1 (*p* = 0.009) were significantly reduced in TAA compared to non-TAA aortic tissue samples. There was no significant difference in MALAT1 (*p* = 0.117) or SMILR (*p* = 0.610) expression between TAA and non-TAA aortic tissue samples.

**Figure 2:**
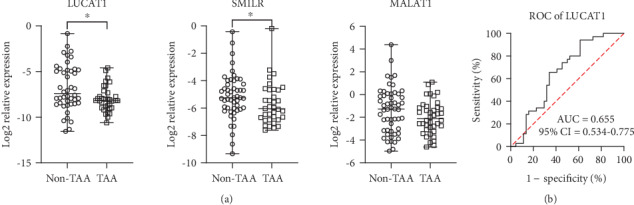
Evaluation of LUCAT1, SMILR, and MALAT1 expression in human blood plasma. (a) Relative expression of LUCAT1, SMILR, and MALAT1 in non-TAA group and TAA patients. (b) Receiver operator characteristic (ROC) curve of LUCAT1 expression in blood plasma for identification of TAA. Whiskers indicate minimum and maximum values; horizontal lines represent median values. ∗ indicates *p* < 0.05. AUC: area under curve; CI: confidence interval.

Expression of LUCAT1 was also significantly reduced (*p* = 0.018) in the blood plasma of TAA patients compared to the non-TAA group (Figure 2(a)). We have also observed reduced SMILR expression (*p* = 0.032) in TAA patients' blood plasma compared to that of the non-TAA group. MALAT1 expression in blood plasma did not differ between the two study groups (*p* = 0.087). Unfortunately, CARMN expression in blood plasma could not be detected using the qRT-PCR method.

A ROC curve analysis for identification of TAA was performed on LUCAT1, MALAT1, and SMILR relative expression levels in blood plasma. An area under curve (AUC) > 0.65 indicates a potential diagnostic value of a certain indicator for the disease [20]. Only LUCAT1 (AUC = 0.654, 95%CI = 0.534‐0.775, *p* = 0.018) represented an ability to discriminate aneurysmal disease in patients' blood plasma (Figure 2(b)).

## 4. Discussion

The thoracic aortic aneurysm has a clinically silent course until development of complications such as aortic dissections. Thus, identification of biomarkers for early diagnostics and clinical course prediction is of great importance and is comparable to the Holy Grail search [2]. Development of thoracic aorta aneurysm is a complex biological process involving smooth muscle cell phenotypic transition. lncRNAs CARMN, LUCAT1, MALAT1, and SMILR are associated with the VSMC phenotypic state in vitro. Our aim was to test if these lncRNAs are dysregulated during TAA formation in clinical patient samples. Thus, we compared expression of these lncRNAs in clinical samples from TAA and non-TAA patients.

CARMN function was first identified in cardiomyocytes as a crucial regulator of cardiac cell differentiation [21]. Ounzain and colleagues demonstrated that CARMN is a super enhancer-associated lncRNA, highly expressed in adult mouse and human heart to maintain cardiac homeostasis and remodeling [21]. CARMN expression is increased in differentiated VSMCs [18], supporting its important role in maintaining a contractile phenotype. We have identified a significant reduction of CARMN in aortic wall samples of TAA patients compared to non-TAA study subjects. Indeed, a high-throughput sequencing analysis has previously identified a significant reduction of CARMN in the aortic wall of thoracic aortic dissection (TAD) patients as well [22]. These results suggest that CARMN plays an important role in the loss of the contractile phenotype of VSMCs and development of TAA. In our study, expression of CARMN could not be detected in patients' blood plasma using the qRT-PCR method. However, increased CARMN expression is observed in peripheral blood nuclear cells (PBNC) of hypertensive patients [23]. Further studies will be needed to test if CARMN expression in patients' PBNCs could be associated with TAA formation.

LUCAT1 was first identified in tumorigenesis as a predictor of poor prognosis in human non-small lung cancer [24]. It is described as a regulator and biomarker for cell cycle progression, proliferation, and metastasis in various cancers [25–28]. A recent article found that downregulation of LUCAT1 inhibits proliferation and promotes apoptosis of cardiomyocytes [29]. LUCAT1 also plays a part in the regulation of apoptosis in smooth muscle cells [30]. Authors show that LUCAT1 depletion is able to increase proliferation and suppress smooth muscle cell apoptosis. In our study, lower expression levels of LUCAT1 in aortic tissue samples from TAA patients could also indicate a regulatory role in VSMC dedifferentiation processes. A significant association between the type of aortic valve and LUCAT1 expression in aortic tissue suggests that it could be a significant player in BAV-associated TAA. Further studies using larger sample sizes will be needed to elucidate the relationship between LUCAT1 and BAV in TAA disease.

LUCAT1 expression in whole blood is relatively abundant compared to other human tissues; therefore, it has great potential to be used as a biomarker for the disease. Decreased expression of LUCAT1 in the blood serum of chronic heart failure patients has been associated with poor prognosis [29] and shows diagnostic potential in identifying diabetic patients with diabetic lung disease [31]. LUCAT1 is also found in blood serum exosomes of hepatocellular carcinoma patients [32]. Our study is the first one to show reduced LUCAT1 expression in the blood plasma of TAA patients and its potential to be used as a disease biomarker in the formation of the disease. A review by Trimarchi and colleagues shows that the RNA signature in a patient's blood could be used for TAA diagnosis and as a possible predictor for aortic expansion with an overall 70%-80% accuracy [33]. Most recent studies show the ability of circulating miRNAs to discriminate TAA patients from controls with AUC values greater than 0.8 in both blood serum [34] and plasma [35] samples. LUCAT1 has a weaker diagnostic potential, but it could be used in combination with other clinical data for diagnosis of TAA.

We did not find significant changes in relative expression of MALAT1 and SMILR in aortic tissue samples between TAA and non-TAA patients. MALAT1 is associated with vascular inflammation as well as endothelial cell function [36] and induces aortic stiffness [37]. Increased SMILR expression is linked to VSMC proliferation in unstable atherosclerotic plaques and in plasma from patients with increased inflammation biomarkers [8, 38]. While VSMC migration is associated with atherosclerosis and is more commonly observed in abdominal aortic aneurysms (AAA), formation of TAA is more often associated with a production of various extracellular proteases and their inhibitors as a result of increased synthetic capabilities of VSMCs. MALAT1 and SMILR could display different mechanisms of VSMC dedifferentiation, which do not play a major part in the development of TAA. Even though SMILR expression in blood plasma was significantly reduced in TAA patients compared to the non-TAA group, it did not pass the necessary threshold for the ability to identify aneurysmal disease in TAA patients.

This study has potential limitations. Firstly, our non-TAA group for tissue analysis consists mainly of CABG patients. Since it is suggested that senescent VSMCs switch to the synthetic phenotype [39], our goal is to match TAA patients and control subjects by age. Heart donors are usually younger than TAA patients; therefore, confounding could rarely be avoided. In addition, the underlying mechanism of LUCAT1 and CARMN in the development of TAA remains unclear and requires further investigation.

## 5. Conclusions

The asymptomatic nature of TAA and lack of medicinal treatment are a driving force behind the biomarker research for the disease. We have shown that LUCAT1 and CARMN might be important players in the formation of TAA. Reduced expression of LUCAT1 shows potential to be used as a minimally invasive biomarker in blood plasma for TAA diagnosis. Further studies will be needed to elucidate the underlying mechanism of this regulatory pathway.

## Figures and Tables

**Table 1:** Clinical characteristics of patients in all study groups.

Characteristic	Tissue			Plasma		
	TAA (*N* = 24)	Non-TAA (*N* = 42)	*p* value	TAA (*N* = 40)	Non-TAA (*N* = 53)	*p* value
Age, yrs median (range)	60 (43-83)	70 (41-86)	0.376	65 (39-87)	70 (41-86)	0.039
Males, *n* (%)	21 (88%)	33 (79%)	0.365	31 (78%)	40 (75%)	0.819
Bicuspid aortic valve, *n* (%)	19 (79%)	1 (2%)	<0.001	18 (45%)	1 (2%)	<0.001
Hypertension, *n* (%)	20 (83%)	36 (86%)	0.067	34 (85%)	49 (92%)	0.251
Aortic valve insufficiency, *n* (%)	18 (75%)	12 (29%)	<0.001	31 (78%)	17 (32%)	<0.001
Aortic valve stenosis, *n* (%)	10 (42%)	2 (5%)	<0.001	13 (33%)	2 (4%)	<0.001
Ascending aortic diameter, mm median (range)	52 (45-65)	35 (28-38)^∗^	<0.001	49 (41-65)	35 (26-39)	<0.001
Aortic annulus, mm median (range)	26 (23-32)	24 (20-28)^∗^	<0.001	27 (22-34)	23 (19-28)	<0.001
Aortic sinus, mm median (range)	48 (38-68)	37 (26-40)^∗^	<0.001	46 (32-74)	37 (26-42)	<0.001

^∗^Data missing from five donors.

## Data Availability

Data will be made available upon request.
